# A systematic literature review of real-world treatment outcomes of small cell lung cancer

**DOI:** 10.1097/MD.0000000000029783

**Published:** 2022-06-30

**Authors:** Michael Stokes, Noami Berfeld, Alicia Gayle, Andrew Descoteaux, Oscar Rohrmoser, April Franks

**Affiliations:** a Evidera, Waltham, Massachusetts, United States; b Evidera, London, United Kingdom; c AstraZeneca, Cambridge, United Kingdom; d AstraZeneca, Gaithersburg, Maryland, United States.

**Keywords:** epidemiology, outcomes, overall survival, small cell lung cancer, treatment patterns

## Abstract

Robust evidence from real-world studies is needed to aid decision-makers and other stakeholders in choosing the best treatment options for patients. The objective of this work was to assess real-world outcomes of treatment strategies for limited- and extensive-stage small cell lung cancer (SCLC) prior to the global introduction of immunotherapies for this disease.

**Methods:**

Searches were conducted in MEDLINE and Embase to identify articles published in English from October 1, 2015, through May 20, 2020. Searches were designed using a combination of Medical Subject Heading (Medline), Emtree (Embase subject headings), and free-text terms such as SCLC. Observational studies reporting data on outcomes of initial treatment strategies in patients with limited- and extensive-stage SCLC were included. Studies with limited sample sizes (<100 patients), enrolled all patients prior to 2010, or did not report outcomes for limited- and extensive-stage SCLC separately were excluded. Data were extracted into a predesigned template by a single researcher. All extractions were validated by a second researcher, with disagreements resolved via consensus.

**Results:**

Forty articles were included in this review. Most enrolled patients from the United States (n = 18 articles) or China (n = 12 articles). Most examined limited-stage (n = 27 articles) SCLC. All studies examined overall survival as the primary outcome. Articles investigating limited-stage SCLC reported outcomes for surgery, chemotherapy and/or radiotherapy, and adjuvant prophylactic cranial irradiation. In studies examining multiple treatment strategies, chemoradiotherapy was the most commonly utilized therapy (56%–82%), with chemotherapy used in 18% to 44% of patients. Across studies, median overall survival was generally higher for chemoradiotherapy (15–45 months) compared with chemotherapy alone (6.0–15.6 months). Studies of extensive-stage SCLC primarily reported on chemotherapy alone, consolidative thoracic radiotherapy, and radiotherapy for patients presenting with brain metastases. Overall survival was generally lower for patients receiving chemotherapy alone (median: 6.4–16.5 months; 3 years, 5%–14.9%) compared with chemotherapy in combination with consolidative thoracic radiotherapy (median: 12.1–18.0 months; 3 years, 15.0%–18.1%). Studies examining whole-brain radiotherapy for brain metastases reported lower median overall survival (5.6–8.7 months) compared with stereotactic radiosurgery (10.0–14.5 months).

**Conclusions:**

Under current standard of care, which has remained relatively unchanged over the past few decades, prognosis remains poor for patients with SCLC.

## 1. Introduction

Worldwide, there are >1800,000 new cases of lung cancer each year.^[1]^ Approximately 15% of new lung cancer cases are small cell lung cancer (SCLC),^[2]^ which is characterized by late diagnosis, poor prognosis, and high frequency of recurrence with existing treatments.^[2–4]^ Lung cancer primarily affects older adults, with a median age at diagnosis of 71 years.^[5]^ Men and women are affected, though incidence rates are higher for men.^[6]^ Based on the National Cancer Institute Surveillance, Epidemiology, and End Results Program data for SCLC in 2017, men and women had age-adjusted incidences of 5.87 and 5.30 cases per 100,000 individuals, respectively.^[7,8]^

There are many prognostic factors to consider for SCLC, including age, presence of metastasis, and performance status.^[9]^ Lung cancer is mainly caused by smoking. For SCLC, the risk is highly elevated for current smokers (odds ratio >100).^[10]^

The Veterans Administration Lung Study Group 2-stage system is typically used to classify SCLC into limited- and extensive-stage diseases. Limited stage refers to cancer that is confined to only 1 lung that can be treated within a single radiation field; extensive stage describes cancer that has spread throughout the lungs or to other organs.^[11]^ The American Joint Committee on Cancer tumor, lymph nodes, metastases (TNM) system, which categorizes disease into stages I to IV,^[11,12]^ is also used. Typically, TNM stage I to III and stage IV map to limited and extensive stages, respectively. Most patients (two-thirds) present with extensive-stage disease, where treatment options are poor and vary depending on disease stage at presentation.

Patients with limited-stage disease are often treated with chemoradiotherapy (CRT) and surgery or radiation for stages I to IIA; chemotherapy (CT) alone has been the standard of care (SOC) in the treatment of extensive-stage disease for the last several decades.^[4]^ According to National Comprehensive Cancer Network (NCCN) guidelines, the preferred primary systemic therapy regimen for limited-stage SCLC includes a platinum agent (such as cisplatin) in combination with etoposide^[9]^; this has been the SOC for >30 years.

For extensive stage, randomized clinical trials (RCTs) recently demonstrated survival benefits with the addition of immunotherapy to CT. In the phase III CASPIAN trial, durvalumab plus platinum–etoposide was associated with a significant improvement in median overall survival (OS; 13.0 vs 10.3 months) over platinum-etoposide alone (hazard ratio [HR] = 0.73; *P* = .0047).^[13]^ Additionally, in the phase III IMpower133 trial, atezolizumab with carboplatin–etoposide showed significant improvement in median OS (12.3 vs 10.3 months; HR = 0.70; *P* = .007) and progression-free survival (5.2 vs 4.3 months; HR = 0.77; *P* = .02) compared with placebo with carboplatin–etoposide.^[14]^ The NCCN guidelines now recommend combining durvalumab or atezolizumab with platinum-based CT as first-line treatment for extensive-stage SCLC.^[9]^

While the efficacy of immunotherapy has been studied in RCTs, data on their real-world (RW) effectiveness in SCLC is limited. To aid decision-makers and other stakeholders in choosing the best treatment options for patients, robust evidence from RW studies is needed. The aim of our review was to provide an overview of RW outcomes, particularly OS, of initial treatment strategies for limited- and extensive-stage SCLC reported in studies published between October 1, 2015, to May 20, 2020.

## 2. Methods

We considered recommendations from the Cochrane Collaboration^[15]^ and Preferred Reporting Items for Systematic Reviews and Meta-Analyses guidelines^[16]^ in the design, conduct, and reporting of the systematic review. Given the nature of this work, specific ethics/institutional review board approval was not applicable.

### 2.1. Search strategies

Searches were conducted to identify RW observational studies examining: (1) the prevalence and incidence of SCLC or (2) treatment patterns and associated outcomes in patients with limited-stage and extensive-stage SCLC. Searches were conducted in MEDLINE (via PubMed.com) and Embase (via Embase.com) to identify original articles published in English from October 1, 2015, to May 20, 2020. This period was chosen to focus on recently published data, consistent with the study’s aim to collect outcomes data from contemporary samples of patients to increase the usability of the data. The studies published prior to October 1, 2015, would have likely included the majority of patients using an enrollment end date that was several years prior to 2015. The searches were conducted on May 20, 2020, and were designed using a combination of Medical Subject Heading (Medline), Emtree (Embase subject headings), and free-text terms included in the abstract or title of a publication. The detailed search strings and corresponding numbers of identified publications are provided in Supplementary Tables 1 and 2, http://links.lww.com/MD/G814.

### 2.2. Study selection

The publications identified by the database searches were deduplicated and screened against predefined inclusion and exclusion criteria (Supplementary Table 3, http://links.lww.com/MD/G814). Studies were included if they reported data on patients with SCLC and assessed disease incidence or prevalence or examined outcomes (including mortality, OS, disease progression, or progression-free survival). Studies were excluded if they had limited sample sizes (<100 patients), enrolled all patients prior to 2010, or did not report OS or progression outcomes, separately, for limited and/or extensive stages. Only full-text papers published in English were included.

Level I abstract screening was conducted by 1 researcher with an initial 15% random sample screened by a second researcher. Upon validation of this initial sample, the accuracy of the included abstracts was deemed acceptable, and no further validation was conducted. Insufficient information in the abstract was not a criterion for exclusion. The full-text articles were retrieved for all abstracts passing the first round of review. Full-text (level II) screening was conducted independently by 2 reviewers. Any discrepancies in decisions to include/exclude studies were resolved by consensus, with input from a third researcher when necessary. For this review, only a subset of studies comparing outcomes of initial treatment strategies was included. Additionally, limited SCLC studies reporting results for TNM stage subgroups only (e.g., stage I or stage III only) were excluded.

### 2.3. Data extraction and synthesis

Data on median OS (months), OS rates at 1, 3, and 5 years, initial treatment strategy, SCLC stage, and data source type were extracted for this study into a predesigned template by a single researcher. All extractions were validated by a second researcher, with disagreements resolved via consensus. Extracted data were grouped according to SCLC stage (limited vs extensive) and synthesized qualitatively. Median estimates (and ranges) for OS statistics were summarized across all included studies by SCLC stage and treatment strategy. Study characteristics, including whether the sample size was ≥500, use of statistical analyses to adjust for confounding effects, and if the study included data on baseline performance status, were also extracted for a simple assessment of study quality. Studies without these characteristics were deemed to be of lower quality compared to studies with some or all of these traits. Note: all included studies used clinical data such as physician diagnoses recorded on medical charts to identify patients with SCLC and classify them by limited and extensive stages.

## 3. Results

### 3.1. Search results

**Figure 1.**
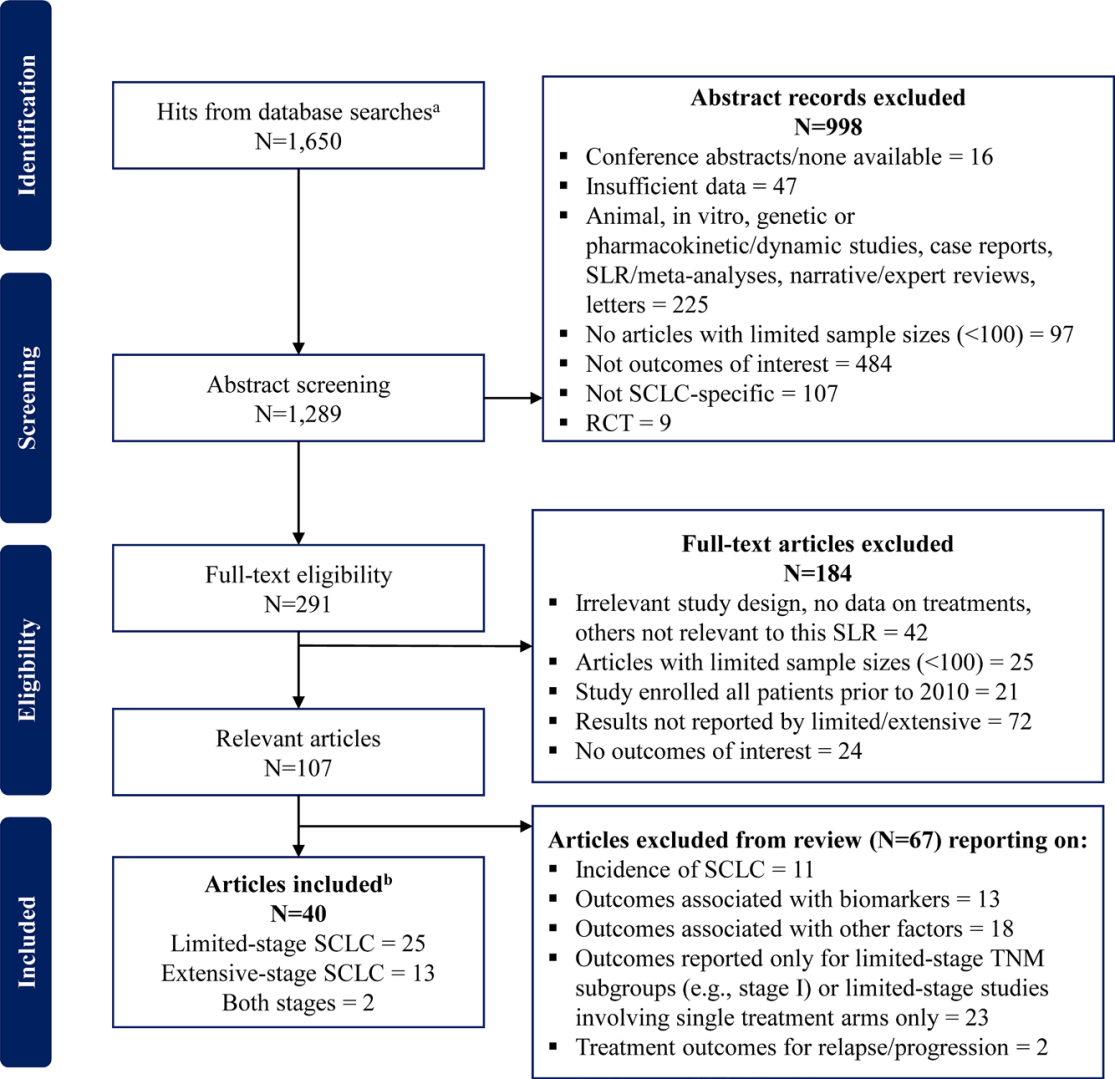
Flow diagram of study selection process. ^a^Combined hits from searches described in Supplementary Tables 1 to 2, http://links.lww.com/MD/G814. ^b^Studies included in the synthesis of results (studies reporting data on outcomes of initial treatment strategies). RCT = randomized controlled trial, SCLC = small cell lung cancer, SLR = systematic literature review.

Database searches identified 1298 unique publications, of which 40 fulfilled the study selection criteria (Fig. 1). Twenty-five publications reported outcomes data for limited stage,^[17–41]^ 13 for extensive stage,^[42–54]^ and 2 for both.^[55,56]^ All studies examined OS as the primary outcome. Studies from China (limited n = 10 extensive n = 6) and the United States (US) (limited n = 12 extensive n = 6) comprised the majority of studies included in the review. Collectively, the studies examining limited- and extensive-stage SCLC enrolled 109,841 and 37,094 patients, respectively.

### 3.2. Study characteristics and quality assessment

#### 3.2.1. Limited stage.

Among the studies examining outcomes of limited-stage SCLC, approximately one-third enrolled patients from large, population-based data sources with a sample size of n ≥ 500.^[17,18,25,27,28,33–36,38,39]^ Of these, nearly all examined patients residing in the US using the secondary National Cancer Database (NCDB) or Surveillance, Epidemiology, and End Results, with 1 examining patients identified from a cancer registry in the Netherlands.^[18]^ The NCDB captures data for 70% of new cancer diagnoses from >1500 Commission on Cancer-accredited program registries in the US. Most studies identified enrolled patients with limited-stage SCLC from single centers with n < 500 patients.^[21,22,26,29–32,37,40,41,55,56]^ Of these, nearly all examined patients in Asia, including 6 in China,^[21,26,30,32,37,40]^ 1 in Japan,^[29]^ and 2 from South Korea.^[22,41]^

Ten studies were identified that compared initial treatment with surgery (± other treatment strategies) to nonsurgical modalities.^[29,32–36,38–40,56]^ Prophylactic cranial irradiation (PCI; yes vs no) was examined in 10 studies; of which 3 looked at PCI following surgery^[27,30,31]^ and 7 examined PCI following CT or CRT.^[19,20,22,26,37,41,55]^ Four studies compared various nonsurgical treatment strategies, including CRT versus CT alone,^[17,24,25]^ CRT vs radiotherapy (RT) alone,^[25]^ and CRT vs palliative CT or RT.^[56]^ Five studies examined various aspects of treatment with CRT, including CRT with radiation delivered twice daily (BID) vs radiation delivered once daily (QD),^[18,21,28]^ and concurrent vs sequential CRT.^[18,23,24]^

Nearly all studies examining limited-stage SCLC employed statistical methods to adjust for differences in baseline characteristics (e.g., age, sex, and stage) between study groups when examining OS. The most commonly used statistical methods included Cox proportional hazard models.^[17–19,23,25–28,30,31,33–36,38,39,41,55,56]^ Matching with propensity scores^[17,29,31,35,57]^ or alternate methods^[32]^ were used in 6 publications. Five studies did not use any statistical adjustment of outcomes,^[20–22,24,40]^ of which only 2 reported that groups were balanced with respect to baseline characteristics.^[21,40]^ Although all studies included cancer stage as a baseline variable, approximately one-half reported data on performance status,^[19–22,24,26,29,31,32,37,41,55,56]^ an important covariate that can influence prognosis. Therefore, residual confounding was likely an issue in many studies.

#### 3.2.2. Extensive stage.

Most publications examining patients with extensive-stage SCLC had <500 patients,^[42–44,46–48,51,53–56]^ with enrollment at single study sites. Of these, most examined patients in China,^[44,46–48,53,54]^ with 2 studies enrolling patients in the US^[42,51]^ and another in Germany.^[43]^ Only one-quarter of studies included data from population-based sources and had a sample n ≥ 500.^[45,49,50,52]^ Among these, all used data from the US NCDB.

Consolidative thoracic radiotherapy (TRT) following CT was compared with CT alone in 7 studies.^[42,46,47,52–54,56]^ PCI was examined in 3 studies,^[44,51,55]^ with 2 comparing PCI versus no PCI^[44,51]^ following CT. Three studies compared whole-brain radiotherapy (WBRT) with stereotactic radiosurgery (SRS) in patients with brain metastases (BM).^[45,48,50]^ One study compared WBRT versus CT alone^[49]^ and another compared outcomes after WBRT in groups with synchronous versus metachronous BM.^[43]^

Statistical analyses adjusting for patient characteristics were used in every study comparing treatment strategies, with most using propensity score matching (PSM) and Cox proportional hazards models^[45–52]^; 5 employed Cox proportional hazard models only^[42–44,54,56]^ and 1 used PSM only.^[53]^ One study was noncomparative and followed patients after PCI only.^[55]^ Most (two-thirds) studies reported data on performance status^[42–44,46–48,53–56]^ and, of these, nearly all enrolled patients outside the US. No publications using the US NCDB included performance status, raising questions as to how well these studies could adequately control for confounding.

### 3.3. Outcomes of limited-stage SCLC

OS according to treatment strategy for limited-stage SCLC is shown in Table 1 and Supplementary Table 4, http://links.lww.com/MD/G814. The median OS exceeded 20 months (range: 18–79 months) in nearly every study examining treatment strategies that included surgery. The highest OS was found in a study of patients (n = 92) undergoing surgery and adjuvant CT with/without RT at West China Hospital.^[40]^ This study enrolled relatively young patients (72% <60 years old), and nearly two-thirds were nonsmokers.

**Table 1:** OS by treatment strategy: limited stage.

Treatment strategy	Median OS (mo)	OS at 1 yr (%)	OS at 5 yr (%)	References
Surgery + CRT	35	86	40	Wei, 2020 (US)^[39]^
Surgery + RT ± CT	-- (32–NR)	85.2 (78.3–92)	43	Kim, 2017 (US)* ^[33]^; Jin, 2018 (US)^[34]^
Surgery + CT	38.6 (18–79)	91 (70–100)	35.3 (28–45)	Wei, 2020 (US)^[39]^; Elegbede, 2020 (Canada)^[56]^; Zhong, 2020 (China)^[40]^; Takenaka (Japan)^[29]^
Surgery without RT ± CT	32 (26–37.1)	82 (75–87)	42 (39–45)	Kim, 2017 (US)^[33]^; Jin, 2018 (US)^[34]^; Chen, 2019 (China)^[32]^
Surgery + CT and/or RT	25.5 (20–31)	73.9 (67.8–80)	34.0 (30–38)	Yang, 2019 (US),^[36]^ Wang, 2020 (US)^[35]^
Surgery without CT	23	59	30	Che, 2018 (US)^[38]^
CRT	21.5 (15–45)	69.3 (60–93)	19.6 (15–45)	Wei, 2020 (US)^[39]^; Elegbede, 2020 (Canada)^[56]^; Chen, 2019 (China)^[32]^; Zhong, 2020 (China)^[40]^; Pezzi, 2018 (US)^[25]^; Corso, 2015 (US)^[17]^; Ohara, 2018 (Japan)^[24]^
QD CRT	27.8 (26–29.5)	90	20.7 (13.3–28.0)	Han, 2015 (China)^[21]^; Damhuis, 2018 (the Netherlands)† ^[18]^
BID CRT	29.2 (27–31.4)	91	21.8 (19.6–23.9)	Han, 2015 (China)^[21]^; Damhuis, 2018 (the Netherlands)† ^[18]^
Sequential CRT	17.5 (17–41)	80 (80–80)	19 (16–42)	Damhuis, 2018 (the Netherlands)† ^[18]^; Manapov, 2016 (Germany)^[23]^; Ohara, 2018 (Japan)^[24]^
CT and/or RT	14 (11–24)	63 (50–75)	19 (10–26)	Yang, 2019 (US)^[36]^; Wang, 2020 (US)^[35]^; Takenaka, 2015 (Japan)^[29]^; Elegbede, 2020 (Canada)^[56]^; Jin, 2018 (US)^[34]^
CT alone	10.3 (6–15.6)	37.5 (19–42)	8.1 (4–15.4)	Wei, 2020 (US)^[39]^; Pezzi, 2018 (US)^[25]^; Corso, 2015 (US)^[17]^; Ohara, 2018 (Japan)^[24]^; Kim, 2017 (US)^[33]^
Surgery + PCI	38 (36–NR)	90 (84–95)	43 (40–59)	Resio, 2019 (US)^[27]^; Yin, 2018 (US)^[31]^; Xu, 2017 (China)^[30]^
Surgery (without PCI)	30 (25.6–60)	82 (78–85)	33.8 (31–50)	Resio, 2019 (US)^[27]^; Yin, 2018 (US)^[31]^; Xu, 2017 (China)^[30]^
CRT‡ + PCI	26 (24–39)	90 (80–96)	32 (15–38)	Farooqi, 2017 (US)^[20]^; Fairchild, 2020 (Canada)^[55]^; Qiu, 2016 (China)^[26]^; Lou, 2017 (China)^[37]^; Koh, 2019 (South Korea)^[22]^; Choi, 2017 (South Korea)^[41]^; Eze, 2017 (Germany)^[19]^
CRT§ (without PCI)	18 (14–42)	82 (45–90)	19 (9–39)	Farooqi, 2017 (US)^[20]^; Qiu, 2016 (China)^[26]^; Koh, 2019 (South Korea)^[22]^; Choi, 2017 (South Korea)^[41]^; Eze, 2017 (Germany)^[19]^

Median estimate (range) for OS statistics across all included studies in category reported.

-- = could not be calculated, BID = twice daily, CRT = chemoradiotherapy, CT = chemotherapy, NR = not reached, OS = overall survival, PCI = prophylactic cranial irradiation, QD = daily, RT = radiotherapy, yr = years.

* Five-year survival rates not reported.

† One-year survival rates not reported.

‡ Use of CRT ranged from 76% to 100%.

§ Use of CRT ranged from 78% to 100%.

There was general agreement across studies that surgery resulted in a significant OS benefit compared with receipt of nonsurgical modalities only. Only a small fraction of patients was eligible for surgery. Among the studies enrolling patients with stage I to III disease, the proportion undergoing surgery was 4.2% to 10.8%.^[24,38,39,56]^ An OS benefit favoring surgery compared with treatment with CT and/or RT was observed in patients in stage I to II (median: 31–34 vs 23–24 months, *P* < .001),^[34,35]^ stage I to III (26-not reached vs 6 months, *P* < .001),^[33]^ and stage IIB–IIIC SCLC (20 vs 14 months, *P* < .001),^[36]^ as well as in patients (stage I–III) with no evidence of receipt of CT (*P* < .001, all).^[38]^ Surgery with CT/CRT conferred a significant survival advantage compared with CT/CRT alone (median OS: 18–79 vs 12–23 months, *P* < .05, all),^[29,39,40,56]^ and relative to palliative CT or RT (40.2 vs 10.7 months, *P* < .001)^[56]^ in stage I to III SCLC. Surgery did not result in a significant survival advantage in patients with stage II to III SCLC^[32]^ or in subgroups with stage IIIB or IIIC.^[36]^

**Figure 2.**
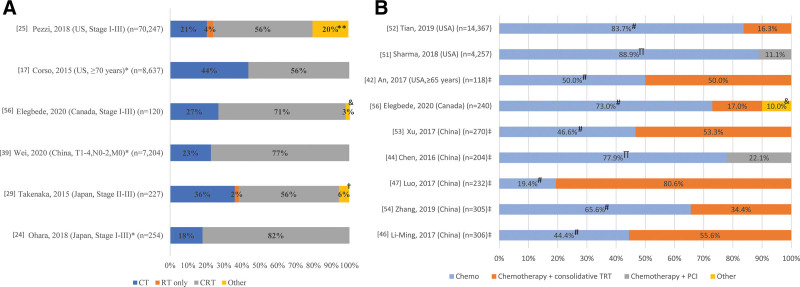
Use of treatment strategies for (A) limited-stage SCLC and (B) extensive-stage SCLC. *Study was limited to patients undergoing chemotherapy. Other category includes: **no chemo or radiotherapy; ^†^surgery only; ^&^not reported in article; ^#^chemotherapy (without consolidative TRT); ^∏^chemotherapy (without PCI); ^‡^enrollment from single study center. Note: [xx], reference number; studies enrolling patients across multiple treatment strategies depicted in figure. CRT = chemoradiotherapy, CT = chemotherapy, PCI = prophylactic cranial irradiation, RT = radiotherapy, TRT = thoracic radiotherapy, US = United States.

**Figure 3.**
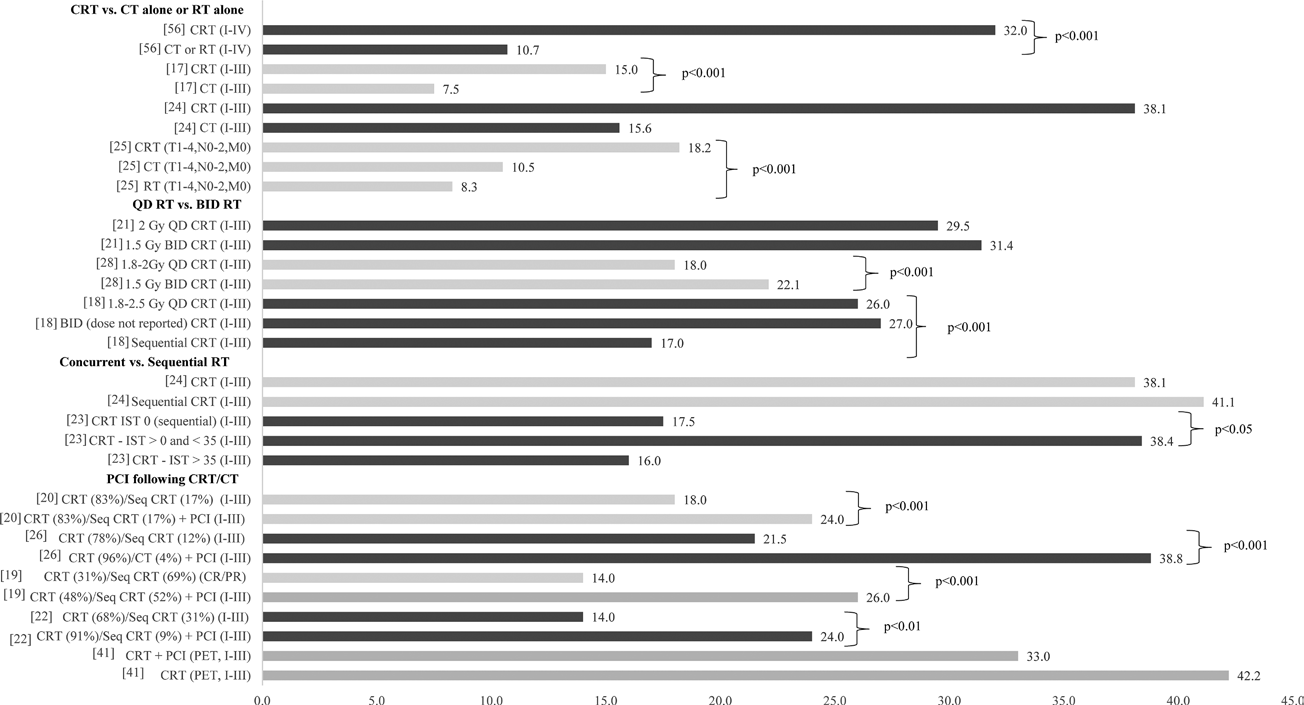
Median OS (months) of limited-stage SCLC nonsurgical treatment strategies. Note: [xx], reference number. BID = twice daily, CR = complete response, CRT = chemoradiotherapy, CT = chemotherapy, IST = interval of simultaneous treatment, PCI = prophylactic cranial irradiation, PET = positron emission tomography, PR = partial response, QD = daily, RT = radiotherapy.

The studies examining patients who did not undergo surgery tended to report higher OS among patients treated with CRT (range: 15–45 months) compared with groups receiving CT and/or RT (11–24 months) and CT alone (6–15.6 months) (Table 1). In studies enrolling patients across multiple treatment strategies, CRT was the most commonly utilized therapy (56%–82%); CT was used in 18% to 44% of patients and RT only in 2% to 4% (Fig. 2A). Four studies compared outcomes of CRT directly with treatment strategies other than surgery^[17,24,25,56]^ (Fig. 3). Significant OS benefits were achieved for patients receiving CRT relative to CT alone (median: 15–32.0 vs 7.5–10.5 months, *P* < .001, all),^[17,24,25]^ RT alone (8.3 months, *P* < .001),^[25]^ and CRT compared with palliative CT or RT (32 vs 10.7 months, *P* < .001).^[56]^ There was significant variability in OS estimates across the studies examining CRT. OS was lowest in groups receiving sequential CRT^[18,23]^ and in a study enrolling only elderly patients (≥70 years old)^[17]^ (Table 1). The highest OS was found in a study enrolling younger patients (64% <60 years old) with high-performance status (93% Karnofsky Performance Status ≥90).^[32]^

Several studies examined whether BID RT with CT had a survival benefit over QD administration^[18,21,28]^ (Fig. 3). Only Schreiber et al^[28]^ reported a statistically significant OS benefit favoring BID administration (median: 22.1 vs 18 months, *P* < .001). However, results should be viewed with caution as the authors did not have access to key variables such as performance status. It is possible that those with worse status were more likely to undergo QD radiation due to better tolerability compared with BID regimens.

Three studies examined the timing of radiation therapy^[18,23,24]^ relative to CT (Fig. 3). Damhuis et al^[18]^ reported a statistically significant OS benefit favoring concurrent CRT versus sequential CRT (median: 26–27 vs 17 months, *P* < .001, all) and Ohara et al^[24]^ did not. Ohara et al did not perform any statistical adjustment to account for differences in baseline characteristics. Additionally, there were notable differences in the age of the cohorts in Ohara et al. For example, patients in the sequential group were much younger compared with the concurrent group. Manapov et al^[23]^ examined CRT and the impact of interval of simultaneous treatment (IST) durations, where IST was defined as the interval in days where CT and RT were applied simultaneously. Although, the sequential (IST = 0) treatment group (median OS: 17.5 months) had significantly lower survival compared with IST >0 to <35 (38.4 months) (*P* < .05), when the IST exceeded 35 days (16 months), no benefit over sequential CRT was realized.

Ten studies examined the benefits of PCI following initial treatment. Median OS ranged from 24 months to not reached (59% survival at 5-year follow-up) in patients receiving PCI. OS was heavily influenced by initial treatment type. In groups receiving surgery,^[27,30,31,53,58]^ 5-year OS rates ranged from 40% to 59% compared with 15% to 38% in patients initially treated with CT or CRT^[19,20,22,26,37,41,55]^ (Table 1). Among the studies of PCI following surgery, a statistically significant OS benefit favoring PCI was observed in stage II (median: 36.4 vs 24.1 months, *P* = .047), stage III (median: 29.3 vs 21.2 months, *P* = .009),^[30]^ stage IIIA (5-year rate: 35.9% vs 18.8%, *P* = .047),^[31]^ and node positive (35% vs 20%, *P* = .008)^[27]^ SCLC, but not in node negative^[27]^ or stage I^30^ SCLC. In the studies comparing PCI with no PCI in patients treated with CRT or CT, there was broad agreement that PCI results in a significant OS benefit^[19,20,22,26]^ (Fig. 3). One exception was in Choi et al,^[41]^ which examined the effect of more accurate staging with positron emission tomography (PET) on the role of PCI. The authors noted that patients who had initial staging with PET achieved long-term survival even without PCI.

### 3.4. Outcomes of extensive-stage SCLC

OS according to treatment strategy for extensive-stage SCLC is summarized in Table 2 and Supplementary Table 5, http://links.lww.com/MD/G814. The median OS for patients treated with CT alone^[42,44,46,47,49,51–54,56]^ ranged from 6.4 to 16.5 months, with median OS exceeding 1 year in only 2 studies.^[44,54]^ In these 2 studies, nearly all (91%)^[54]^ or all patients^[44]^ receiving platinum-based CT responded to treatment. The lowest OS was observed in a study enrolling patients ≥75 years old with BM (n = 238) from the NCDB.^[52]^ Studies that primarily enrolled patients with 1 metastatic site^[42]^ and those that excluded patients with disease progression^[47]^ or nonresponse to CT^[44,51]^ tended to report higher OS compared with studies enrolling primarily patients with polymetastases^[53]^ or BM,^[49]^ or studies without criteria requiring response to CT.^[46,52]^ In the publications reporting information on CT regimens,^[42,44,46,47,51,53,54,56]^ nearly all patients (84%–100%) received platinum-based therapy. In the studies (3 from China and 1 from the US) reporting receipt of specific agents, 86% to 100% of patients enrolled received cisplatin-etoposide or carboplatin-etoposide regimens; 3 publications did not report information on the specific CT regimen received.^[49,51,52]^ Median OS for patients receiving CT and consolidative TRT (range: 12.1–18.0 months)^[42,46,47,52–54,56]^ or CT and PCI (range: 12.0–16.5 months)^[42,44,51,55]^ was ≥12 months (Table 2).

**Table 2:** OS by treatment strategy: extensive stage.

Treatment strategy	Median OS (mo)	OS at 1 yr (%)	OS at 3 yr (%)	Studies
CT alone	11.1 (6.4–16.5)	44 (15–65)	10 (5–14.9)	Tian, 2019 (US)^[52]^; An, 2017 (US)^[42]^; Renz, 2019 (US)^[49]^; Li-Ming, 2017 (China)^[46]^; Zhang, 2019 (China)^[54]^; Luo, 2017 (China)^[47]^; Chen, 2016 (China)^[44]^
CT + consolidative TRT	17.7 (12.1–18)	65 (50.5–80)	15 (15–18.1)	Tian, 2019 (US)^[52]^; An, 2017 (US)^[42]^; Elegbede, 2020 (Canada)^[56]^; Li-Ming, 2017 (China)^[46]^; Zhang, 2019 (China)^[54]^; Luo, 2017 (China)^[47]^; Xu, 2017 (China)^[53]^
CT + PCI	13.9 (12–16.5)	64.0 (42–70)	13.8 (0–18.1)	An, 2017 (US)^[42]^; Sharma, 2018 (US)^[51]^; Fairchild, 2020 (Canada)^[55]^; Chen, 2016 (China)^[44]^
WBRT*	7.1 (5.6–8.7)	30 (18–41)	4 (4–16)	Bernhardt, 2017 (Germany)^[43]^; Ni, 2020 (China)^[48]^; Robin, 2008 (US)^[50]^; Renz, 2019 (US)^[49]^; Jiang, 2019 (US)^[45]^
WBRT + boost*	13.6 (9.3–17.9)	53.5 (39–68)	16.5 (6–27)	Ni, 2020 (China)^[48]^; Jiang, 2019 (US)^[45]^
SRS*	12.9 (10–14.5)	49 (42–54)	19 (7–31)	Jiang, 2019 (US)^[45]^; Robin, 2018 (US)^[50]^; Ni, 2020 (China)^[48]^

Median estimate (range) across included studies reported.

CT = chemotherapy, PCI = prophylactic cranial irradiation, SRS = stereotactic radiosurgery, TRT = thoracic radiotherapy, WBRT = whole-brain radiotherapy, yr = years.

* Treatment given to patients with brain metastases.

**Figure 4.**
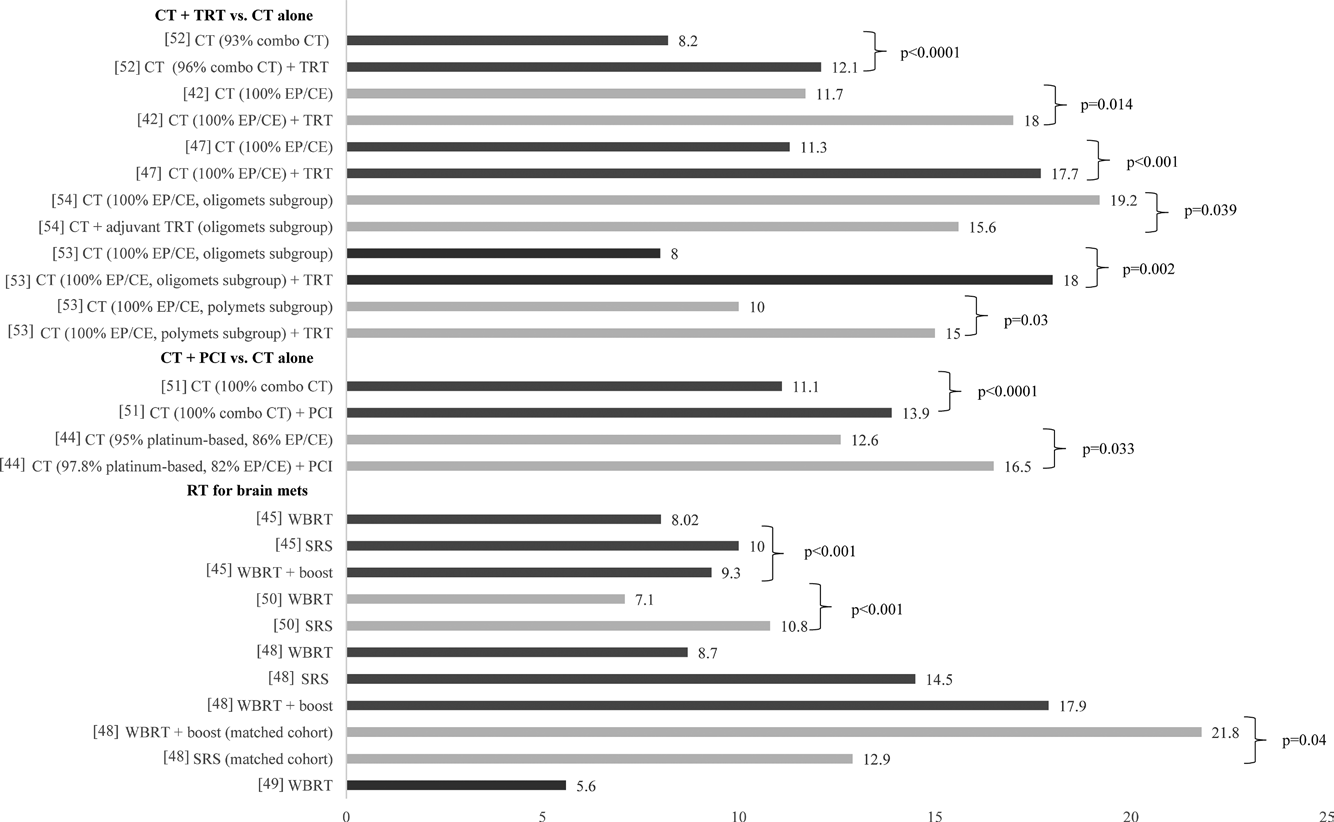
Median OS (months) of extensive-stage SCLC treatment strategies. Note: [xx], reference number. BM = brain metastases, CE = carboplatin-etoposide, CT = chemotherapy, EP = etoposide-platinum, PCI = prophylactic cranial irradiation, RT = radiotherapy, SRS = stereotactic radiosurgery, TRT = thoracic radiotherapy, WBRT = whole-brain radiotherapy.

There was general agreement across studies showing that consolidative TRT following CT resulted in superior OS outcomes compared with CT alone^[42,47,52,53]^ (Fig. 4). However, Zhang et al^[54]^ found a significant benefit only for patients with oligometastases (median OS: 19.2 vs 15.6 months, *P* = .039), but not for those with brain/liver/multimetastasis. This contrasts with results from Xu et al,^[53]^ which demonstrated a significant benefit in separate subgroups with oligometastases (2-year OS: 25.2% vs 12.7%, *P* = .002) and polymetastases (10.0% vs 6.8%, *P* = .030). Two studies enrolling patients without BM concluded that PCI following CT was beneficial compared with CT alone. In a study using the NCDB that excluded patients with OS <6 months as a proxy for lack of response to CT, median OS was 13.9 months for PCI versus 11.1 months for those who did not receive PCI (*P* < .001).^[51]^ Similarly, in a single-center study enrolling patients who had an initial response to CT, PCI significantly prolonged median OS from 12.6 to 16.5 months (*P* = .033).^[44]^ Another single-center study did not find a significant benefit favoring PCI.^[42]^ However, this study enrolled only patients ≥65 years old and failed to exclude those with BM. Data from large US population-based studies indicated that only 16.3% and 11.1% of patients receive TRT and PCI, respectively (Fig. 2B).^[51,52]^ Although, in single-center studies, TRT treatment rates were much higher (34.4%–80.6%).

In studies examining patients presenting with BM, median OS was consistently higher with SRS (range: 10.0–14.5 months)^[45,48,50]^ compared with WBRT (5.6–8.7 months)^[43,45,48–50]^ (Table 2). Three studies compared WBRT to SRS,^[45,48,50]^ 2 of which also assessed WBRT and boost^[45,48]^ (Fig. 4). A significant survival benefit for SRS compared with WBRT was observed in 2 studies using the NCDB; 1 enrolling patients from 2010 to 2014 (median OS: 10.8 vs 7.1 months, *P* < .001)^[50]^ and the other from 2004 to 2013 (10.0 vs 8.2 months, *P* < .001).^[45]^ Jiang et al^[45]^ reported that SRS led to significantly better survival compared with WBRT and boost (10.0 vs 9.3 months, *P* < .001). Ni et al,^[48]^ however, found that WBRT and boost significantly improved OS relative to SRS in the PSM analysis (21.8 vs 12.9 months, *P* = .040), but not in the overall sample. One study found that WBRT led to a modest increase in survival (1.9 vs 1.2 months, *P* < .0001) compared with no WBRT in the subgroup not treated with CT.^[49]^

## 4. Discussion

This review provides insights into RW treatment patterns and outcomes for patients with limited- or extensive-stage SCLC. Despite the limitations associated with RW data, many studies comparing treatment strategies generated evidence consistent with findings from RCTs and treatment guidelines. To our knowledge, this is one of the first reviews in SCLC summarizing the RW evidence separately for limited and extensive stages.

### 4.1. Limited stage

NCCN guidelines recommend surgical resection with lobectomy as the preferred radical therapy in T1-2N0 SCLC only,^[9]^ which represents a small proportion of patients with SCLC. Consistent with this recommendation, we found that the surgery is infrequent in limited-stage SCLC (4.2%–10.8%). In recent years, investigators have shown that a multimodality approach including surgery may have benefits, particularly for stage I (cancer confined to 1 lung only) and II (cancer confined to 1 lung and nearby lymph nodes) patients, despite a lack of evidence from RCTs.^[59–61]^ We identified 10 publications comparing multimodal therapy, including surgery to approaches using CT and/or RT only in limited-stage SCLC. Nearly all studies demonstrated significant survival benefits favoring treatment approaches including surgery; however, no improvement in OS following surgery was seen in patients with stage II–III SCLC.

For patients receiving CRT, median OS ranged from 15 to 45 months. Publications examining treatment with CT and/or RT (11–24 months) or CT alone (6–16 months) reported lower median OS relative to CRT. Concurrent CRT is the current SOC for limited-stage SCLC. Data from clinical trials found that concurrent CRT, where RT starts within the first or second cycle of CT, is more effective compared with sequential CRT.^[62–64]^ Our review identified several RW studies examining the sequence and timing of RT with mixed results. Two showed a significant OS benefit favoring concurrent versus sequential CRT,^[18,23]^ whereas the study by Ohara et al^[24]^ did not. However, no statistical adjustment of outcomes was conducted in Ohara et al, despite notable differences between groups in patient characteristics.

BM commonly occurs in patients with SCLC. More than 50% will develop BM within 2 years.^[65]^ Therefore, PCI is considered the SOC in patients with limited-stage SCLC. PCI was associated with longer OS in nearly all studies identified examining PCI following surgery. However, a survival benefit was not observed in subgroups with node negative or stage I SCLC. In the studies of patients comparing PCI with no PCI following CT or CRT,^[19,20,22,26]^ all except Choi et al^[41]^ observed a significant OS benefit for PCI. The authors noted that patients with an initial PET staging evaluation achieved long-term survival even without PCI.

### 4.2. Extensive stage

Consistent with treatment guidelines,^[9,66]^ most extensive-stage patients included in this review received CT only. Most studies reported a median OS ≤12 months for patients treated with CT alone, indicating the poor prognosis of extensive-stage SCLC. The most common type of CT regimen was platinum-based, used in 84% to 100% of patients with extensive-stage disease, which aligns with the established SOC for the past several decades. Recently, atezolizumab and durvalumab have demonstrated improvement in survival when combined with platinum-based regimens for first-line treatment of extensive-stage SCLC.^[13,14]^ However, we did not identify any RW studies examining these novel immunotherapies, which is likely a reflection of the review’s study period and the fact that it usually takes several years before records of these treatments show up in retrospective databases and cancer registries.

Historically, RT was reserved for palliation in extensive-stage SCLC.^[67]^ The fact that many patients had recurrent or progressive intrathoracic disease led to clinical trial investigations and other observational studies examining the role of consolidative TRT in this population. In the first RCT examining consolidative TRT following CT, TRT led to significant OS improvements.^[68]^ This review identified 6 studies comparing CT with or without consolidative TRT. A statistically significant OS benefit favoring TRT was reported in nearly all studies. Consolidative TRT is recommended in patients with complete response or good response following CT. Three studies examining PCI in extensive-stage SCLC were identified that reported mixed results. Two studies showed a significant OS improvement with PCI,^[44,51]^ however, no OS benefit was observed with PCI in a study enrolling only elderly patients.^[42]^ Mixed results in RCTs examining PCI in extensive-stage SCLC have also been reported. An RCT conducted by the European Organization for Research and Treatment of Cancer found that PCI improved survival,^[69]^ while another from Japan found no improvement compared with routine surveillance magnetic resonance imaging and treatment of asymptomatic BM.^[70]^ According to the NCCN guidelines, PCI is not recommended in patients with poor performance status or impaired neurocognitive function.

NCCN guidelines state that WBRT should typically be used as treatment in patients who present with BM. Although WBRT is considered the SOC for BM, the optimal therapeutic approach remains controversial.^[71]^ Two studies included in our review found that SRS was associated with significantly longer survival compared with WBRT.^[45,50]^ Although the studies included in this review were observational and the data cannot be used to definitively conclude that survival would be improved with one approach over another, the data suggest that SRS may be appropriate and lead to favorable outcomes for certain patient subgroups. An RCT is being conducted to compare SRS to WBRT.^[72]^

### 4.3. Limitations

The searches for this systematic review were conducted in MEDLINE and Embase. Therefore, any publications that were relevant but not included in these databases were not captured in our search. As for any review, the current study is limited by the available literature and the possibility of publication bias. The systematic literature review included studies from a relatively short period of only ~4.5 years. However, the size of the literature on SCLC is very large and the time period was sufficiently long to identify multiple studies reporting outcomes for all the SOC treatment strategies across various geographic regions. In addition, the review period allowed for the identification of studies enrolling patients from more recent time periods increasing the usability of the data. Validation of level I abstract screening was only conducted for a random sample (15%) of abstracts, and some accuracy may have been compromised compared to double-screening. As a substantial proportion of the publications were retrospective chart reviews or secondary database analyses, the data from the studies also had important limitations. For example, many publications did not report information on performance status, an important variable affecting prognosis. Although many studies used statistical methods to control for imbalances in patient characteristics, residual confounding was likely an issue. Additionally, in cases where the survival data was extracted from a graph, the information is inherently less precise.

## 5. Conclusions

The RW evidence collected as part of this systematic review reveals a limited number of available SCLC treatment options. Overall, the recent RW evidence was in alignment with established treatment recommendations and clinical trial results for SCLC. In limited stage, higher OS rates may be achieved with treatment approaches including surgery (vs without), particularly for stage I SCLC and, in patients ineligible for surgery, concurrent CRT is preferable to sequential CRT and approaches using CT or RT alone. Additionally, PCI following CRT provides an OS benefit compared to CRT alone in limited stage. In extensive stage, consolidative TRT following CT results in higher OS compared to CT alone. Further study is needed to determine if PCI following CT prolongs survival in extensive stage and whether SRS is more effective compared to WBRT in patients who present with BM. Under the current SOC, which has remained relatively unchanged over the past few decades, prognosis remains dismal for many patients with SCLC. Recently approved immunotherapies may be the beginning of a new era where significant improvements in outcomes are observed. Our findings will enable healthcare decision-makers and other stakeholders to put RCT data on emerging therapies into context with RW outcomes achieved under the current SOC.

## Acknowledgments

This study was funded by AstraZeneca. Copy-editing and medical writing support was provided by Michael Grossi, of Evidera. Lisa Mather, of Evidera, contributed to abstract/full-text screening and data extraction.

## Author contributions

Conceptualization – MS, NB, AD.

Data curation – MS, NB, AD.

Formal analysis – MS, NB, AD.

Funding acquisition – OR, AF.

Investigation – MS, NB, AD.

Methodology – MS, NB, AD.

Project administration– MS, NB, AD.

Resources– MS, NB, AD.

Software– MS, NB, AD.

Supervision– MS, NB, AD,OR, AF, AG.

Validation– MS, NB, AD.

Visualization – MS, NB, AD.

Writing – original draft – MS, NB, AD.

Writing – review and editing – MS, NB, AD, OR, AF, AG.

## Supplementary Material


