# Supplementary material for: A systematic literature review of real-world treatment outcomes of small cell lung cancer
Source: Medicine (Baltimore). 2022 Jun 30;101(26):e29783. doi: 10.1097/MD.0000000000029783 (PMC9239604; doi:10.1097/MD.0000000000029783)
Supplement: Supplementary file 1 [file medi-101-e29783-s001.pdf]

Supplementary Table 1. Search Strings Used for Searches in Medline

Search #	Search Terms	Hits[a]
1	exp Small Cell Lung Carcinoma/	3,952
2	(small-cell lung cancer or small-cell lung carcinoma or small-cell lung cancers or small-cell lung carcinomas or oat-cell carcinoma or oat-cell cancer or oat-cell carcinomas or oat-cell cancers).ti,ab.	72,692
3	(non-small or nonsmall).ti,ab.	65,616
4	2 not 3	12,364
5	Limit 4 to "pubmed not medline"	942
6	((oat-cell or small-cell lung) and (Incidence or Prevalence or Demography or age or sex or Mortality or Overall Survival or Natural history or Disease progression or Prognosis or Comorbidity or ECOG)).ti.	2,154
7	6 not (non-small or nonsmall).ti.	225
8	((oat-cell or small-cell lung) adj3 (Incidence or Prevalence or Demography or age or sex or Mortality or Overall Survival or Natural history or Disease progression or Prognosis or Comorbidity or ECOG)).ab.	731
9	8 not (non-small or nonsmall).ti,ab.	108
10	((Lung cancer or lung carcinoma) and (Incidence or Prevalence or Demography or age or sex or Mortality or Overall Survival or Natural history or Disease progression or Prognosis or Comorbidity or ECOG)).ti.	4,975
11	10 not (non-small or nonsmall).ti.	3,009
12	Limit 11 to "pubmed not medline"	236
13	1 or 5 or 7 or 9 or 12	5,269
14	13 not (animals/ not humans/)	5,247
15	(case report or case series or two cases or three cases or four cases or woman or man or child or adolescent or female or male or boy or girl or infant).ti.	837,703

Search #	Search Terms	Hits[a]
16	Exp case report/ or case report$.ti. or case report$.jn.	2,156,290
17	14 not (15 or 16)	4,416
18	(Ephemera or "Introductory Journal Article" or News or "Newspaper Article" or Editorial or Comment or Overall or Letter).pt. or exp In Vitro Techniques/ or exp Immunochemistry/ or (commentary or editorial or comment or letter or mice or rat or mouse or animal or murine or xenograft$ or in vitro or assay).ti. or cell line$.ti,ab.	4,673,034
19	Review.pt. not (systematic or meta$).ti,ab.	2,200,404
20	exp Quality Control/ or quality control.ti,ab. or Models, Theoretical/ or theoretical study.ti,ab. or methodology study.ti,ab.	239,121
21	Exp Randomized Controlled Trial/ or exp Random Allocation/ or exp Double-Blind Method/ or exp Cross-over studies/ or (rct or cct or controlled trial or clinical trial or single or double or double-blind or randomized or randomized or cross-over or crossover or placebo or phase).ti,ab. or trial.ti.	3,605,972
22	17 not (18 or 19 or 20 or 21)	2,319
23	Limit 22 to English language	2,091
24	Limit 23 to yr=”2015 -Current”	1,115
25	Limit to abstract	1,095
26	(2015 jan$ or 2015 feb$ or 2015 mar$ or 2015 apr$ or 2015 may or 2015 jun$ or 2015 july or 2015 aug$ or 2015 sep$ or 2015 spring).dp.	573,598
27	25 not 26	**1,028**

Note: MeSH subject headings are noted with a “/” following the term e.g., Small Cell Lung Carcinoma/ [a] Searches conducted on May 20, 2020.

Supplementary Table 2. Search Strings Used for Searches in Embase

Search #	Search Terms	Hits[a]
1	Exp *small cell lung cancer/	4,787
2	(small-cell lung cancer or small-cell lung carcinoma or small-cell lung cancers or small-cell lung carcinomas or oat-cell carcinoma or oat-cell cancer or oat-cell carcinomas or oat-cell cancers).ti,ab.	114,234
3	(non-small or nonsmall).ti,ab.	104,958
4	2 not 3	17,804
5	((oat-cell or small-cell lung) and (Incidence or Prevalence or Demography or age or sex or Mortality or Overall Survival or Natural history or Disease progression or Prognosis or Comorbidity or ECOG)).ti.	3,385
6	5 not (non-small or nonsmall).ti.	394
7	((oat-cell or small-cell lung) adj3 (Incidence or Prevalence or Demography or age or sex or Mortality or Overall Survival or Natural history or Disease progression or Prognosis or Comorbidity or ECOG)).ab.	1,131
8	7 not (non-small or nonsmall).ti,ab.	181
9	4 or 6 or 8	17,831
10	Limit 9 to exclude medline journals	1,655
11	1 or 10	5,830
12	11 not ((exp animal/ or nonhuman/) not exp human/)	5,604
13	(case report or case series or two cases or three cases or four cases or woman or man or child or adolescent or female or male or boy or girl or infant).ti.	916,886
14	Exp case report/ or case report$.ti. or case report$.jn.	2,496,010
15	animal experiment/ or cancer cell culture/ or nonhuman/ or diagnostic test accuracy study/	6,704,952
16	12 not (13 or 14 or 15)	4,180

Search #	Search Terms	Hits[a]
17	(Books or Chapter or Editorial or Letter or Note or Short Survey).pt. or exp in vitro study/ or exp immunochemistry/ or (commentary or editorial or comment or letter or mice or rat or mouse or animal or murine or xenograft$ or in vitro or assay).ti. or cell line$.ti,ab.	9,948,837
18	Review.pt. not (systematic or meta$).ti,ab.	2,160,287
19	exp quality control/ or quality control.ti,ab. or theoretical model/ or theoretical study.ti,ab. or methodology study.ti,ab.	515,400
20	exp randomized controlled trial/ or exp randomization/ or exp double blind procedure/ or exp crossover procedure/ or exp placebo/ or (rct or cct or controlled trial or clinical trial or single or double or double-blind or randomized or randomized or cross-over or crossover or placebo or phase).ti,ab. or trial.ti.	4,602,286
21	16 not (17 or 18 or 19 or 20)	1,650
22	Limit 21 to English language	1,350
23	Limit 22 to yr="2015 -Current"	1,151
24	Limit 23 to (article or article-in-press)	630
25	Limit 24 to abstracts	437
26	(2015 jan$ or 2015 feb$ or 2015 mar$ or 2015 apr$ or 2015 may or 2015 jun$ or 2015 july or 2015 aug$ or 2015 sep$ or 2015 spring).dp.	180
27	25 not 26	626
28	Remove duplicates from 27	**622**

Note: Emtree subject headings are noted with a "/" following the term e.g., small cell lung cancer/ [a] Searches conducted on May 20, 2020.

Supplementary Table 3. Inclusion and Exclusion Criteria for Article Selection

Variable	Inclusion Criteria	Exclusion Criteria
Population	Studies including patients with SCLC	Studies including patients with non-SCLC or lung without reporting outcomes separately for SCLC
Intervention and comparators	None	None
Outcomes	Prevalence and incidence of SCLC<br>Mortality<br>OS<br>Disease progression<br>Progression-free survival	OS or progression outcomes data from patients with SCLC overall, without stratification by extensive and/or limited disease
Study design	Retrospective or prospective observational studies<br>Database- and registry-based studies	Animal, in vitro, or genetic studies<br>RCT<br>Systematic reviews/meta-analyses<br>Case reports or case series
Study size	N ≥ 100	N < 100
Time period	Study published between October 2015 and May 2020	Study published before or after time period<br>Studies enrolling all patients prior to 2010
Language	English	Languages other than English
Type of publications	Full-text articles	Conference abstracts

Abbreviations: OS = overall survival; RCT = randomized controlled trials; SCLC = small cell lung cancer

Supplementary Table 4. Overall Survival by Treatment Strategy: Limited Stage

Author, Year	Stage	Treatment Details	Number Patients	Median OS (months)	OS 1-year, %	OS 2-year, %	OS 3-year, %	OS 5-year, %	Data Source
**Surgery + CRT**									
Wei 2020 (US)[39]	T1-4,N0-2,M0	Surgery + CRT	267	35	*86*	*59*	*49*	*40*	SEER
**Surgery + CT**									
Wei 2020 (US)[39]	T1-4,N0-2,M0	Surgery + CT	296	37	*86*	*62*	*51*	42.6	SEER
Elegbede 2020 (Canada)[56]	I-IV	Surgery + CT	8	40.2	*100*	*100*	*70*	*45*	Glans-Look database
Takenaka 2015 (Japan)[29]	II-III	Surgery + CT (75%)/CRT (18%)	44	*18.0*	*70.0*	*40.0*	*35.0*	28.0	Single cancer center
Zhong 2020 (China)[40]	I-III	Surgery + CT ± RT	50	79	96	80	*70*	28	Single-center hospital
**Surgery + RT ± CT**									
Kim 2017 (US)[33]	I-III	Surgery + RT ± CT	92	--	78.3	*57*	NR	NR	SEER
Jin 2018 (US)[34]	I-II	Surgery + RT ± CT	154	34	*92*	*60*	*46*	42.7	SEER

Author, Year	Stage	Treatment Details	Number Patients	Median OS (months)	OS 1-year, %	OS 2-year, %	OS 3-year, %	OS 5-year, %	Data Source
**Surgery + CT and/or RT**									
Yang 2019[36]	IIB-IIIC	Surgery + CT and/or RT	998	*20*	67.8	41.5	32.6	*30*	SEER
Wang 2020[35]	I-II	Surgery + CT and/or RT	475	*31*	*80*	*56*	48	*38*	SEER
**Surgery without RT ± CT**									
Kim 2017 (US)[33]	I-III	Surgery, No RT ± CT	84	26	74.7	*55*	NR	NR	SEER
Jin 2018 (US)[34]	I-II	Surgery, No RT ± CT	387	32	*82*	*60*	*47*	38.9	SEER
Chen 2019 (China)[32]	I-III	Surgery + Adjuvant CT 81.2%	69	37.1	*87*	60.1	*52*	45	Single-center hospital
**Surgery without CT**									
Che 2018 (US)[38]	I-III	RT 1.5%	520	*23*	*59*	*45*	*38*	*30*	SEER
**CRT**									
Wei 2020 (US)[39]	T1-4,N0-2,M0	CRT	1,012	20	*68*	*43*	*30*	20.1	SEER
Elegbede 2020 (Canada)[56]	I-IV	CRT	71	32.0	*90*	*60*	*45*	*30*	Glans-Look database

Author, Year	Stage	Treatment Details	Number Patients	Median OS (months)	OS 1-year, %	OS 2-year, %	OS 3-year, %	OS 5-year, %	Data Source
Chen 2019 (China)[32]	I-III	CRT	69	45.00	*93*	63.8	*57*	45	Single-center hospital
Pezzi 2018 (US)[25]	T1-4,N0-2,M0	CRT	38,979	18.20	60	40	30	19	NCDB
Corso 2015 (US, ≥70 years)[17]	I-III	CRT	4,862	*15.00*	*60*	*30*	22	*15*	NCDB
Zhong 2020 (China)[40]	I-III	CRT	102	23.0	70.6	48	*30*	16.7	Single-center hospital
Ohara, 2018 (Japan)[24]	I-III	CRT	175	38.10	*90*	*70*	*52*	36	Multi- cancer center
		Sequential CRT	33	41.1	*80*	*65*	*50*	41.6	Multi- cancer center
Han 2015 (China)[21]	I-III	QD CRT	80	29.5	*90*	43	*40*	13.3	Single-center hospital
		BID CRT	63	31.4	*91*	49	*42*	19.6	Single-center hospital
	I-III	QD CRT	414	26	NR	52	NR	28	Cancer registry

**Author, Year**	**Stage**	**Treatment Details**	**Number Patients**	**Median OS (months)**	**OS 1-year, %**	**OS 2-year, %**	**OS 3-year, %**	**OS 5-year, %**	**Data Source**
Damhuis 2018 (the Netherlands)[18]		BID CRT	407	27.00	NR	54	NR	31	Cancer registry
		Sequential CRT	667	17	NR	33	NR	16	Cancer registry
Schreiber 2015 (US)[28]	I-III	BID CRT	2,821	22.1	*75*	*47*	*35*	23.9	NCDB
		QD CRT	22,224	*18*	*70*	*39*	*28*	*17.9*	NCDB
Manapov 2016 (Germany)[23]	I-III	CRT - IST > 0 and < 35	20	38.40	*85*	*75*	*50*	*25*	Multi-center
		CRT - IST > 35	51	16	*56*	*30*	*19*	*15*	Multi-center
		CRT IST 0 (sequential)	111	17.5	*80*	*34*	*25*	*19*	Multi-center
**CT and/or RT**									
Yang 2019 (US)[36]	IIB-IIIC	CT and/or RT	2,994	*14*	60.6	35.1	24.8	*19*	SEER
Wang 2020 (US)[35]	I-II	CT and/or RT	475	*22.5*	*63*	*45*	34	*25*	SEER
Takenaka 2015 (Japan)[29]	II-III	CT (20%), RT (3%), CRT (78%)	44	*12.0*	*50.0*	*25.0*	*15.0*	11.0	Single cancer center
Elegbede 2020 (Canada)[56]	I-IV	CT or RT	38	10.7	*70*	*15*	*10*	*10*	Glans-Look database
Jin 2018 (US)[34]	I-II	RT alone (± CT)	1,032	24	*75*	*50*	*37*	25.90	SEER
**RT alone**									

Author, Year	Stage	Treatment Details	Number Patients	Median OS (months)	OS 1-year, %	OS 2-year, %	OS 3-year, %	OS 5-year, %	Data Source
Pezzi 2018 (US)[25]	T1-4,N0-2,M0	RT alone	2,465	8.3	42	19	15	10	NCDB
**CT alone**									
Wei 2020 (US)[39]	T1-4,N0-2,M0	CT alone	1,347	10	*40*	*17*	*10*	6.1	SEER
Pezzi 2018 (US)[25]	T1-4,N0-2,M0	CT alone	14,383	10.5	42	19	15	10	NCDB
Corso 2015 (US, ≥70 years)[17]	I-III	CT alone	3,775	*7.5*	*35*	*10*	6.3	*4*	NCDB
Ohara, 2018 (Japan)[24]	I-III	CT alone	46	15.6	*19*	*17*	*15*	15.4	Multi-cancer center
Kim 2017 (US)[33]	I-III	No RT (± CT)	647	6	27.2	*15*	NR	NR	SEER
**No CT**									
Che 2018 (US)[38]	I-III	RT 6.5%	4,260	*2*	*20*	*10*	*7*	*7*	SEER
**Surgery + PCI**									
Resio, 2019 (US)[27]	I-III	Surgery + CT + PCI	202	NR	*95*	*80*	*74*	59	NCDB

Author, Year	Stage	Treatment Details	Number Patients	Median OS (months)	OS 1-year, %	OS 2-year, %	OS 3-year, %	OS 5-year, %	Data Source
Yin, 2018 (US)[31]	II-III	Surgery + CT/CRT + PCI	61	*40*	84	*69*	58	43	Single-center hospital
Xu, 2017 (China)[30]	I-III	Surgery + CT + PCI	115	36	*90*	62	*55*	40	Single-center hospital
**Surgery (no PCI)**									
Resio, 2019 (US)[27]	I-III	Surgery + CT	657	*60*	*85*	*70*	*60*	50	NCDB
Yin, 2018 (US)[31]	II-III	Surgery + CT/CRT	55	*30*	82	*59*	43	33.8	Single-center hospital
Xu, 2017 (China)[30]	I-III	Surgery + CT	234	25.6	*78*	50	*42*	31	Single-center hospital
**CT and/or RT + PCI**									
Farooqi, 2017 (US)[20]	I-III	CRT (83%)/Seq CRT (17%) + PCI	364	*24*	*88*	57	*38*	*25*	Single cancer center
Qiu, 2016 (China)[26]	I-III	CRT (96%)/CT(4%) + PCI	185	39	96	*69*	53	30	Single-center hospital
Koh, 2019 (South Korea)[22]	I-III	CRT (91%)/Seq CRT (9%) + PCI	160	*24*	*85*	59	*40*	*38*	Single-center hospital

Author, Year	Stage	Treatment Details	Number Patients	Median OS (months)	OS 1-year, %	OS 2-year, %	OS 3-year, %	OS 5-year, %	Data Source
Choi, 2017 (South Korea)[41]	I-III	CRT 100% (PET) + PCI	52	33	*90*	*72*	*41*	38	Single-center hospital
Eze, 2017 (Germany)[19]	I-III	CRT (48%)/Seq CRT (52%) + PCI	71	26	*80*	*45*	*30*	*15*	Multi-cancer center
Fairchild, 2020 (Canada)[55]	I-III	CRT (76%)/CT(24%) + PCI	72	*24*	*92*	*60*	*45*	*32*	Single cancer center
Lou, 2017 (China)[37]	I-III	CRT (95%)/CT(5%) + PCI	188	28	95	69	55	NR	Single-center hospital
**CT and/or RT (no PCI)**									
Farooqi, 2017 (US)[20]	I-III	CRT (78%) + Seq CRT (22%)	294	*18*	*88*	44	*27*	*17*	Single cancer center
Qiu, 2016 (China)[26]	I-III	CRT (78%)/CT (12%)	214	22	82	*50*	35	25	Single-center hospital
Koh, 2019 (South Korea)[22]	I-III	CRT (68%)/Seq CRT (31%)	190	*14*	*78*	38	*22*	*19*	Single-center hospital
Choi, 2017 (South Korea)[41]	I-III	CRT 100% (PET)	91	42	*90*	*72*	*59*	39	Single-center hospital

Author, Year	Stage	Treatment Details	Number Patients	Median OS (months)	OS 1-year, %	OS 2-year, %	OS 3-year, %	OS 5-year, %	Data Source
Eze, 2017 (Germany)[19]	I-III	CRT (31%)/Seq CRT (69%) (CR/PR)	NR	14	*45*	*36*	*10*	*9*	Multi-cancer center

Note: italics indicates that data were extracted from survival curves

Abbreviations: BID = twice daily; CR = complete response; CRT = chemoradiotherapy; CT = chemotherapy; IST = IST = interval of simultaneous treatment; NCDB = National Cancer Database; NR = not reached; OS = overall survival; PCI = prophylactic cranial irradiation; PR = partial response; QD = daily; RT = radiotherapy; SEER = Surveillance, Epidemiology, and End Results; US = United States

Supplementary Table 5. Overall Survival by Treatment Strategy: Extensive Stage

**Author, Year**	**Treatment Detail**	**Number Patients**	**Median OS (months)**	**OS 1-year, %**	**OS 2-year, %**	**OS 3-year, %**	**OS 5-year, %**	**Data Source**
**CT alone**								
Tian 2019 (US)[52]	93% combo CT	12,019	8	23	*8*	*6*	*5*	NCDB
An 2017 (US, ≥65 years)[42]	100% EP/CE	59	11.7	*50*	14.90	14.90	*2.00*	Single study site
Li-Ming 2017 (China)[46]	100% Platinum-based CT	136	NR	NR	10.3	NR	NR	Single study site
Zhang 2019 (China)[54]	100% EP/CE	200	16.5	*65.0*	*20*	*12.0*	*5*	Single study site
Luo 2017 (China)[47]	100% EP/CE	45	11.3	27.3	*6*	*5*	NR	Single study site
Xu 2017 (China)[53]	100% EP/CE	22	*8*	*15*	12.7	NR	NR	Single study site
Sharma 2018 (US, OS>6 months)[51]	100% combo CT	472	11.1	44	11.5	*5.00*	NR	NCDB
Chen 2016 (China, CR/PR only)[44]	95% Platinum-based, 86% EP/CE	159	12.6	*52*	*22*	*10*	NR	Single study site
Elegbede 2020 (Canada)[56]	84% Platinum-based CT	128	8.7	45	10	10	8	Glans-Look database
Renz 2019 (US, ≥75 years + BM)[49]	100% CT	238	6.4	*22*	*8*	NR	NR	NCDB

Author, Year	Treatment Detail	Number Patients	Median OS (months)	OS 1-year, %	OS 2-year, %	OS 3-year, %	OS 5-year, %	Data Source
**CT alone or with TRT**								
An 2017 (US, ≥65 years)[42]	100% EP/CE alone or with TRT	49	13	*64*	20.3	5.1	*5*	Single study site
**CT + consolidative TRT**								
Tian 2019 (US)[52]	96% combo CT + TRT	1,461	12.1	50.5	*22*	*15*	*10*	
An 2017 (US, ≥65 years)[42]	100% EP/CE + TRT	59	18	*70*	38.1	18.10	*10.00*	Single study site
Li-Ming 2017 (China)[46]	100% Platinum-based + TRT	170	NR	NR	21.4	NR	NR	Single study site
Zhang 2019 (China)[54]	100% EP/CE + TRT	105	17.8	*78.0*	*35.0*	*18.0*	*10*	Single study site
Luo 2017 (China)[47]	100% EP/CE + TRT	45	17.7	57.9	*25*	*15*	NR	Single study site
Xu 2017 (China)[53]	100% EP/CE + TRT	22	*18*	*65*	25.2	NR	NR	Single study site
Elegbede 2020 (Canada)[56]	84% Platinum-based CT + TRT	40	13.4	80	30	15	13	Glans-Look database
**Chemotherapy + PCI**								
An 2017 (US, ≥65 years)[42]	100% EP/CE alone or with TRT + PCI	43	13.4	*64*	24.9	18.1	5	Single study site

Author, Year	Treatment Detail	Number Patients	Median OS (months)	OS 1-year, %	OS 2-year, %	OS 3-year, %	OS 5-year, %	Data Source
Sharma 2018 (US, OS >6 months)[51]	100% combo CT + PCI	472	13.9	61.20	19.80	*10.00*	NR	NCDB
Fairchild 2020 (Canada)[55]	CT (65%)/RT (29.1%) + PCI	97	12	*42*	*15*	*0.00*	NR	Tertiary cancer center
Chen 2016 (China, CR/PR only)[44]	97.8% Platinum-based, 82% EP/CE	45	16.5	*70*	*35*	*17.5*	NR	Single study site
**WBRT**								
Renz 2019 (US, ≥75 years + BM)[49]	100% CT + WBRT 30 Gy	576	5.6	*18*	*8*	NR	NR	NCDB
Jiang 2019 (US, BM)[45]	83% CT + WBRT	13,657	8.02	*30*	*8*	*4*	*1*	NCDB
Bernhardt 2017 (Germany, BM)[43]	100% CT + WBRT 30 Gy	229	6	*21*	*7*	*4*	*1*	Single hospital center
Ni 2020 (China, BM)[48]	71% CT + WBRT 25-45 Gy	140	8.7	*41*	*18*	*16*	NR	3 study sites
Robin 2018 (US, BM)[50]		5,752	7.1	*30*	*8*	NR	NR	NCDB

Author, Year	Treatment Detail	Number Patients	Median OS (months)	OS 1-year, %	OS 2-year, %	OS 3-year, %	OS 5-year, %	Data Source
**WBRT + boost**								
Ni 2020 (China, BM)[48]	70% CT + WBRT 25-45 Gy + boost	79	17.9	*68*	*41*	*27*	NR	NCDB
Ni 2020 (China, BM, matched cohort)[48]	71% CT + + WBRT 25-45 Gy + boost	37	21.8	*78*	*48*	*28*	NR	NCDB
Jiang 2019 (US, BM)[45]	83.7% CT	578	9.3	*39*	*11*	*6*	*2*	NCDB
**SRS**								
Jiang 2019 (US, BM)[45]	81% CT	487	10	*42*	*12*	*7*	*3*	NCDB
Robin 2018 (US, BM)[50]		200	10.8	*47*	*15*	NR	NR	NCDB
Ni 2020 (China, BM)[48]	66% CT + WBRT 10.5-20.5 Gy	44	14.5	*54*	*41*	*31*	NR	NCDB
Ni 2020 (China, BM, matched cohort)[48]	68% CT + WBRT 10.5-20.5 Gy	37	12.9	*51*	*30*	NR	NR	NCDB

Note: italics indicates that data were extracted from survival curves

Abbreviations: BM = brain metastases; CE = carboplatin/etoposide; CR = complete response; CT = chemotherapy; EP = etoposide/platinum; NCDB = National Cancer Database; NR = not reached; OS = overall survival; PCI = prophylactic cranial irradiation; PR = partial response; SRS = stereotactic radiosurgery; TRT = thoracic radiotherapy; US = United States; WBRT = whole-brain radiotherapy
